# Deep Semantic Segmentation of Angiogenesis Images

**DOI:** 10.3390/ijms24021102

**Published:** 2023-01-06

**Authors:** Alisher Ibragimov, Sofya Senotrusova, Kseniia Markova, Evgeny Karpulevich, Andrei Ivanov, Elizaveta Tyshchuk, Polina Grebenkina, Olga Stepanova, Anastasia Sirotskaya, Anastasiia Kovaleva, Arina Oshkolova, Maria Zementova, Viktoriya Konstantinova, Igor Kogan, Sergey Selkov, Dmitry Sokolov

**Affiliations:** 1 Information Systems Department, Ivannikov Institute for System Programming of the Russian Academy of Sciences (ISP RAS), 109004 Moscow, Russia; 2 Department of Immunology and Intercellular Interactions, Federal State Budgetary Scientific Institution, Research Institute of Obstetrics, Gynecology, and Reproductology Named after D.O. Ott, 199034 St. Petersburg, Russia

**Keywords:** angiogenesis, endothelial cells, deep learning, semantic segmentation

## Abstract

Angiogenesis is the development of new blood vessels from pre-existing ones. It is a complex multifaceted process that is essential for the adequate functioning of human organisms. The investigation of angiogenesis is conducted using various methods. One of the most popular and most serviceable of these methods in vitro is the short-term culture of endothelial cells on Matrigel. However, a significant disadvantage of this method is the manual analysis of a large number of microphotographs. In this regard, it is necessary to develop a technique for automating the annotation of images of capillary-like structures. Despite the increasing use of deep learning in biomedical image analysis, as far as we know, there still has not been a study on the application of this method to angiogenesis images. To the best of our knowledge, this article demonstrates the first tool based on a convolutional Unet++ encoder–decoder architecture for the semantic segmentation of in vitro angiogenesis simulation images followed by the resulting mask postprocessing for data analysis by experts. The first annotated dataset in this field, AngioCells, is also being made publicly available. To create this dataset, participants were recruited into a markup group, an annotation protocol was developed, and an interparticipant agreement study was carried out.

## 1. Introduction

The formation of blood vessels in an adult organism occurs due to the process of angiogenesis, which is the process of vessel formation from existing ones. In addition to growth and development, angiogenesis includes renewal, regeneration, and an increase in the branching of blood vessels [1,2,3]. Angiogenesis is one of the most important processes that take place in the human body, without which its adequate function is impossible. Blood vessels arose in evolution to allow haematopoietic cells to perform immune surveillance, to supply oxygen and nutrients, and to dispose of waste. Vessels also produce instructive signals for organogenesis in a perfusion-independent manner [2,4,5,6].

Angiogenesis occurs in the adult organism both in normal and pathological conditions [6]. Normal physiological processes involving angiogenesis are the female reproductive cycle, placentation, wound healing, tissue regeneration, and hair renewal. However, angiogenesis also contributes to pathological conditions [7]. Suboptimal vascular growth can lead to a stroke, myocardial infarction, peptic ulcers, and neurodegenerative diseases. Abnormal growth or remodelling of blood vessels underlies tumour formation, inflammation, pulmonary hypertension, and blindness [4,8].

All vessels are internally lined with endothelial cells (ECs), which form a monolayer and are in a state of rest [2,4]. In a stable vessel of a healthy organism, ECs form a cobblestone monolayer and are in a relatively inactive state. Such a resting phenotype is maintained until ECs pick up an angiogenic signal that causes significant changes in their behaviour [9]. In response to tissue damage or lack of oxygen and nutrients, or in pathological conditions, ECs become activated, and their further behaviour, particularly, the formation of new vessels, depends on the cellular microenvironment and cytokines secreted by both ECs and the microenvironment cells [4].

To date, two types of angiogenesis have been described: branching and nonbranching [2,10]. Nonbranching angiogenesis is the process of increasing the length of pre-existing vessels, and branching angiogenesis is the formation of vessels by lateral capillary budding or the connection of existing vessels [2,4]. Different types of angiogenesis underlie different pathological processes. The existing interest in the study of vessel formation mechanisms is based on the possibility of creating test kits, therapies, and treatments of different pathologies.

The study of angiogenesis is carried out using various experimental models including the observation of the formation of vessels in various thin organs and structures of lower animals or developing embryos of birds, the transparent chamber method, and the study of the growth of vessels in the cornea of the eye of rodents and *Danio rerio* fish [11]. However, one of the most popular and simple methods for studying angiogenesis in vitro is the short-term culture of ECs on Matrigel, which is a gelatinous protein mixture obtained from Engelbreth-Holm-Swarm mouse sarcoma cells. Endothelial cells migrate, differentiate, and form capillary-like structures on Matrigel in the presence of different mediators. The formation of tube-like vessels under these conditions can be used to assess compounds that either inhibit or stimulate angiogenesis [12]. This Matrigel assay is quick and easy to perform and also allows in vitro modelling of endothelial cell behaviour, including survival, apoptosis, and the steps leading to capillary formation and invasion. It is also important for investigating the effects of drugs or small molecules on angiogenesis in vitro before they are developed into clinical therapies [13]. As a result of studies using this method, researchers can obtain illustrative images of various stages of angiogenesis. By processing microphotographs, they can estimate the length and number of formed vessels and the number and area of “cell nodes”, which are clusters of cells that give points of growth to the vessels. These parameters are important for understanding the stage of the angiogenesis process and its mechanism. Establishing the mechanisms of angiogenesis and understanding the behaviour of ECs as a result of the action of a mediator is important for the further development of therapies. However, the evaluation of the obtained parameters causes a number of difficulties for the researcher: a manual analysis of a large number of images, which requires significant time and labour contribution, as well as the subjectivity of the image analysis.

Currently, these kinds of images are analysed as follows: images are preprocessed to correct uneven illumination using the polynomial method of background correction [14], which allows for the creation of a clearer contrast between the cells and the background [15], then, using contour detection and hierarchical image segmentation [16], the cells are segregated, followed by skeletonization. Ten basic parameters of the network structure are quantified by the skeleton: branches, closed networks, nodes, network areas, network structures, triple-branched nodes, quad-branched nodes, total branch length, average branch length, and the branch-to-node ratio [17,18]. The disadvantage of this approach is the sensitivity and accuracy at the segmentation stage, e.g., when analysing, images with insufficient illumination; defective images taken with a poor-quality microscope or obtained out of the plane of focus; and images that contain various kinds of objects, such as debris or single cells (of which there are many in the early stages of angiogenesis), which do not contain important information.

Despite good results in medical image analysis being obtained through deep learning methods [19,20,21], to the best of our knowledge, there has not been any research done on the semantic segmentation of blood vessel images obtained by in vitro angiogenesis simulation. In this paper, we propose segmenting the ECs of blood vessels on the image using the Unet++ architecture [22], then postprocessing to extract quantities. This approach allows for the division of objects formed by ECs into two categories: nodes and tubes (Supplementary Annotation Protocol (Schemes S1 and S2)), which leads to an increase in the derived parameters from the image, such as tube length, tube coverage area, and node area (Supplementary Figure S1).

Neural networks typically contain a huge number of trainable parameters and require a large number of images for better performance. Annotating an appropriate number of images from different stages of angiogenesis can be very challenging, especially when a strict definition of objects and structures is required, and the lack of labelling data in this area remains a significant obstacle to numerical image analysis. The creation of labelled data requires a specialist’s involvement, although even experienced experts may show some inconsistency in formally defining objects. Annotations have to be made on many images, but since significant differences can be observed even within the same laboratory, which can affect the learning process of the network architecture substantially, an annotation protocol (AP) has been developed and its correctness has been tested with interparticipant agreement (Section 4.3).

To the best of our knowledge, AngioCells is the first open data collection that enables an automated picture analysis of the angiogenesis process, and we also publish it here. The dataset is available at vessels.ispras.ru (accessed on 27 December 2022) under a Creative Commons Attribution 4.0 International license [23].

## 2. Results

### 2.1. Encoder Selection

In 2015, network encoder–decoder architectures were introduced [24,25], including Unet [26]. The decoder’s purpose is to convert full-resolution input feature maps from low-resolution encoder features for pixelwise classification. The novelty of such architectures is how the decoder upsamples lower-resolution input feature maps. In particular, the decoder uses the pooling indices calculated in the max-pooling step of the corresponding encoder to perform nonlinear upsampling. The Unet architecture consists of a sequence of nonlinear processing layers (encoder) and a corresponding set of decoder layers followed by a pixelwise classifier. Ronneberger et al. [26] added skip connections to the encoder–decoder image segmentation networks, e.g., SegNet, which increased the model’s accuracy and solved the issue of vanishing gradients, much like in image recognition [27] and keypoint detection [28]. Each encoder typically comprises a few convolutional layers with batch normalization, a ReLU nonlinearity, nonoverlapping max-pooling, and subsampling. The max-pooling indices in the encoding sequence are used in the decoder to upsample the sparse encoding caused by the pooling procedure. This type of network architecture has proven itself in image segmentation competitions such as satellite image analysis [29], medical image analysis [30,31], and others [32].

It is well known that to train the network without overfitting, the dataset must be relatively large, comprising millions of images. In most cases, data sets for image segmentation consist of a maximum of thousands of images; in our case, it was 275 annotated images, since the manual preparation of masks is a very expensive procedure. There is a method to train Unet on a relatively small training set. As a rule, the classification model (without the last dense layers), trained on ImageNet as a feature extractor to build a segmentation model [33], is taken as an encoder. Thus, the training procedure can be performed on the untrained multiple layers of the decoder (sometimes only for the last layer) to take into account the features of the data set. This training method is described in this subsection.

**Figure 1:**
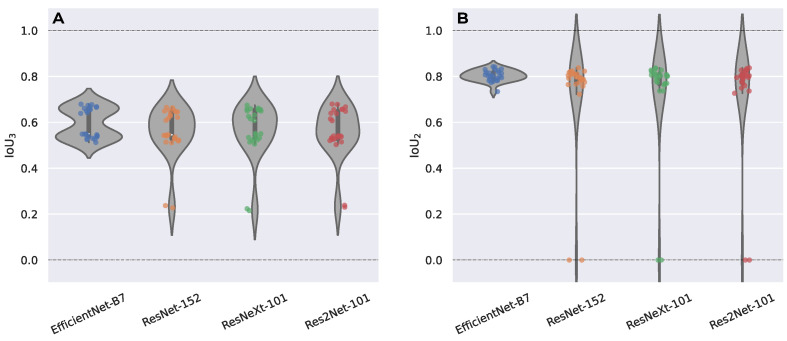
Prediction scores for the validation data; each dot represents performance for *k*th model of the 25-fold cross-validation; 4 encoders were considered: EfficientNet-B7, ResNeXt-101, ResNet-152, and Res2Net-101. (**A**) Performance by IoU${}_{3}$ metric. The Wilcoxon signed-rank test: median performance of Unet with EfficientNet-B7 was greater than for the model with ResNeXt-101, ResNet-152, and Res2Net-101 with a confidence level of 0.95. (**B**) Performance by IoU${}_{2}$ metric. The Wilcoxon signed-rank test: the median performance of Unet with EfficientNet-B7 was greater than for the model with Res2Net-101, ResNet-152 with a confidence level of 0.95.

As an encoder in our Unet neural network, we considered the following classifiers: EfficientNet-B7 [34], ResNeXt-101 [35], ResNet-152 [36], and Res2Net-101 [37]. The selection of such a set of networks was due to the high accuracy in image classification on ImageNet dataset competitions and the availability of weights in the public domain. Figure 1 presents the result of training networks on all data types for a *k*-fold cross-validation. To analyse the statistical significance of the proposed architectures with different encoders, the nonparametric statistical Wilcoxon signed-rank test [38] was used, with a typical rule being a requirement that k>20 [39], so *k* was chosen to be 25. Using the IoU${}_{3}$ (and IoU${}_{2}$) metric on the validation samples, a pair of architectural performances was employed for the statistical test. A pair of models was tested using the one-sided test: the null hypothesis (H${}_{0}$) corresponded to the median of the first model of the pair being less than the median of the second model of the pair. The significance level set for the test was α=0.05 (or a confidence level of 0.95). If the *p*-value of the test was less than the significance level α, then the null hypothesis was rejected in favour of an alternative hypothesis (H${}_{a}$): the median of the first model out of the pair was greater than the median of the second model out of the pair (further, the first model had greater performance than the second model). If the *p*-value of the test was greater than the significance level α, then no assumptions were made. The results of this comparison were as follows: Unet with EfficientNet-B7 had a greater performance (IoU${}_{3}$) than the model with ResNeXt-101, ResNet-152, and Res2Net-101 with a confidence level of 0.95 (Figure 1A); Unet with EfficientNet-B7 had a greater performance (IoU${}_{2}$) than Res2Net-101 and ResNet-152 with a confidence level of 0.95 (Figure 1B). Later in this paper, an architecture with EfficientNet-B7 as an encoder was used, as it had a greater performance by the IoU${}_{3}$ metric compared to architectures from other encoders and a greater performance by the IoU${}_{2}$ metric compared to two other architectures. Supplementary Figure S2 shows Figure 1 with performance scores.

### 2.2. Optimization Loss Function

The choice of a loss function is extremely important in deep learning complex architectures for the semantic segmentation of images, as the resulting network performance depends on it. To enhance the outcomes of their datasets, researchers have been experimenting with various domain-specific loss functions since 2012 [40]. The most commonly used loss function for the task of image segmentation is the pixelwise cross-entropy (CE) loss [41]. Therefore, it was selected for the encoder in Section 2.1 for our Unet.

The loss value should rise monotonically as more false positives and negatives are expected. S. Asgari Taghanaki et al. showed that, for large objects, almost all considered functions followed this assumption; however, for small objects, only some functions penalized monotonically more for larger errors [41]. Therefore, the focal loss (FL) function was selected to achieve a greater stability when training on both large (nodes and backgrounds) and small (tubes) objects. In our case, the proportion of tubes, nodes, and backgrounds were $\frac{N_{tubes}}{N}≃0.05$, $\frac{N_{nodes}}{N}≃0.26$, and $\frac{N_{background}}{N}≃0.69$, respectively, where $N_{c}$ is the number of pixels marked as class *c* and *N* is the total number of pixels in the dataset. For the case of a binary segmentation, the focal loss for class *c* can be written in the following form [42]:

(1) $$\mathrm{FL}\left(p_{t}\right)=-\alpha_{t}{\left(1-p_{t}\right)}^{\gamma }\log\left(p_{t}\right),$$

where γ⩾0 is a tunable focusing parameter. $\alpha_{t}=1-\frac{N_{c}}{N}$ was used to mitigate class imbalance. For notational convenience, $p_{t}$ was defined as:

(2) $$p_{t}=\left\{\begin{array}{cc} p & \;\mathrm{if}\;y=c\\ 1-p & \;\mathrm{otherwise}.\; \end{array}\right.$$

In the above, *y* specifies the ground-truth class and p∈[0,1] is the model’s estimated probability for the class with label y=c, where c∈{0,1,2} (background, nodes, tubes). Extending the focal loss to the multiclass case yields the sum of the FL for each class. We found γ=0.5 to work best in our experiments (Supplementary Figure S3). The resulting model on the test on all data showed the following performance: IoU${}_{2}=0.803\pm 0.016$ and IoU${}_{3}=0.643\pm 0.014$. As can be seen, this approach improved the performance of the small object (tubes) detection network due to the modulating factor ${\left(1-p_{t}\right)}^{\gamma }$ [40], which confirmed the feasibility of using the focal loss in subsequent experiments.

### 2.3. Architecture Selection

**Figure 2:**
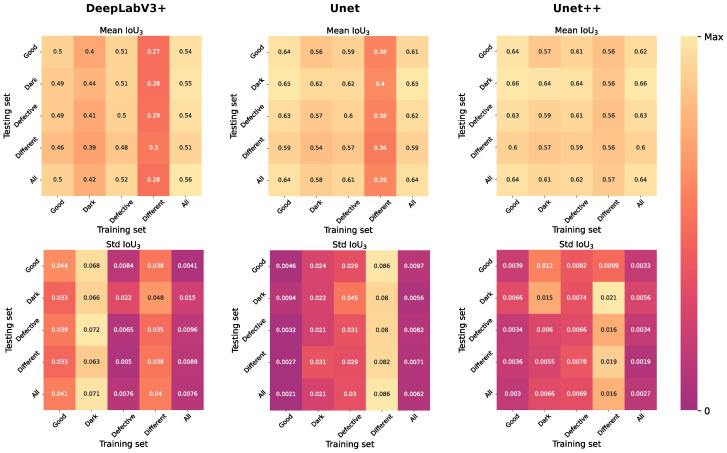
Performanceon test data belonging to different groups based on models trained on different training data. The first row contains the mean values of IoU${}_{3}$. The second row contains standard errors of mean IoU${}_{3}$. Each column corresponds to one of the selected network architectures—DeepLavV3+, Unet, Unet++.

**Figure 3:**
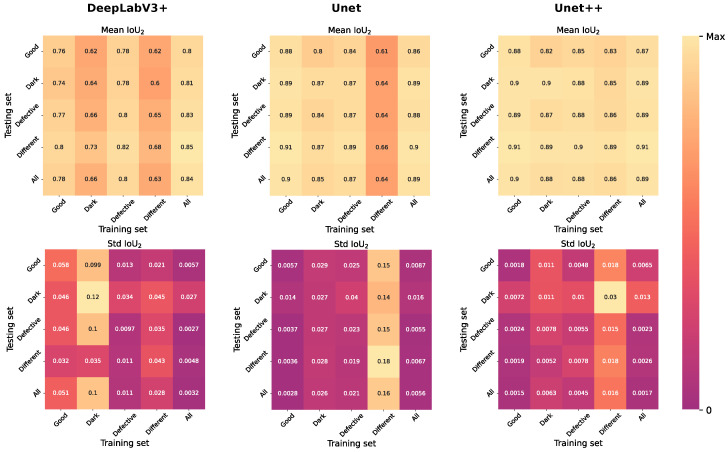
Performanceon test data belonging to different groups based on models trained on different training data. The first row contains the mean values of IoU${}_{2}$. The second row contains standard errors of mean IoU${}_{2}$. Each column corresponds to one of the selected network architectures—DeepLavV3+, Unet, Unet++.

In this study, in order to achieve the best network quality, two modern architectures different from Unet were also considered—DeepLabV3+ [43] and Unet++ [22]. These two methods, as well as Unet, were initialized using the EfficientNet-B7 encoder pretrained on ImageNet, selected in Section 2.1. For a more detailed study, each of the models was trained and tested on different data types (Table 1). The results of training and testing are demonstrated in Figure 2 and Figure 3. As you can see, Unet++ demonstrated not only the best quality according to the IoU${}_{3}$ and IoU${}_{2}$ metrics, but also the greatest stability, judging by the standard deviation charts. Note the Unet architecture and DeepLabV3+, trained on the different data groups, were much inferior in quality to the Unet++ architecture. In this regard, the final architecture of the neural network was Unet++ based on the pretrained classifier EfficientNet-B7. Table 2 shows the performance results for an all groups–all groups (training–test) pair. You can also notice that training on all data was more stable than training on one type of subdatasets. Most likely, this was due to both the large number of labelled images and the variety of data obtained: various stages of angiogenesis, photos with a defect and uneven lighting when shooting, and high-quality data obtained from the final stage of angiogenesis.

### 2.4. Fine-Tuning

As discussed in Section 2.1, training a deep convolutional neural network (CNN) from scratch is challenging, especially in medical applications where annotated data are scarce and expensive. An alternative to full training is transfer learning, where a network that has been trained on a large dataset is tuned for another application. When the new data set is small, the recommended approach to training the network is to leave the first layers of the network untrained (frozen layers) and subject the last layers to training (unfrozen layers) [44]. A CNN’s first layers are demonstrated to represent more low-level features, whereas deeper layers are shown to identify more semantic and high-level features [45]. Therefore, training only the deepest layers (decoder) assumes that the basic characteristics of the datasets (associated with the encoder) are similar and the more specific characteristics of the datasets (associated with the decoder) need to be adjusted to get acceptable results in a different application. This assumption may not be true in some medical applications, such as microscope images of blood vessels, due to their specificity compared to data from ImageNet.

M. Amiri et al. showed that, due to their dataset-specific patterns, the encoder training when freezing the decoder exhibited better performance [46]. Therefore, this section looks at improving network performance by unfreezing the encoder blocks one-by-one (fine-tuning) from the beginning (first block) to the end (tenth block). By “block”, we mean a set of layers that have a total number of parameters equal to ∼6M so that it is possible to divide the encoder (∼63.8M) into 10 blocks. The process of fine-tuning is as follows:

1. The model obtained in Section 2.3 was taken, all layers were frozen, the first block was unfrozen, and the network was trained using a fivefold cross-validation;
2. The best performing network (IoU${}_{3}$) from the previous experiment was taken, all layers were frozen, the next block was unfrozen, and the network was trained using a fivefold cross-validation;
3. Step 2 was repeated until the network with a fully fine-tuned encoder was obtained.

**Figure 4:**
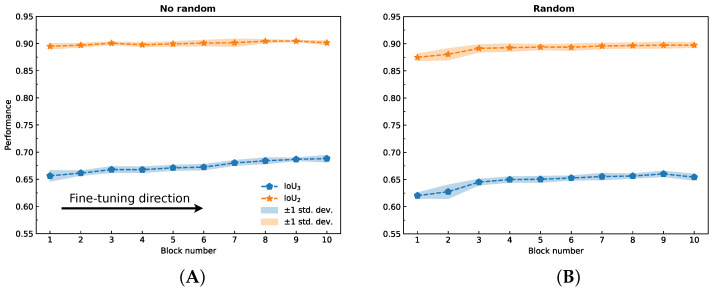
Average IoU${}_{3}$ and IoU${}_{2}$ scores depend on the number of trainable blocks in the EfficientNet-B7 encoder. The arrow shows the direction of the fine-tuning blocks: from the first to the last. (**A**) With fine-tuning, the block weights are not reset. Achieved performance: IoU${}_{3}=0.703\pm 0.005$ and IoU${}_{2}=0.83\pm 0.01$. (**B**) With fine-tuning, the weights of trainable block are reset. Achieved performance: IoU${}_{3}=0.538\pm 0.004$ and IoU${}_{2}=0.804\pm 0.010$.

The obtained result is shown in Figure 4A. A similar experiment was carried out where the unfreezing of the following block was also accompanied by a reset of the weights to random values (Figure 4B), but this approach showed a lower performance: IoU${}_{3}=65.5\pm 0.5$% versus IoU${}_{3}=65.93\pm 0.11$%. This procedure was also performed in the reverse direction for the Unet architecture (from the 10th block to the 1st block in the encoder), but in this case, there was almost no noticeable improvement (Supplementary Figure S4). For comparison, the model performance results for each experiment are shown in Table 3. Therefore, to solve this problem, we propose to use the Unet++ neural network architecture based on the EfficientNet-B7 encoder, followed by a fine-tuning procedure (proposed method).

### 2.5. Qualitative Results

**Figure 5:**
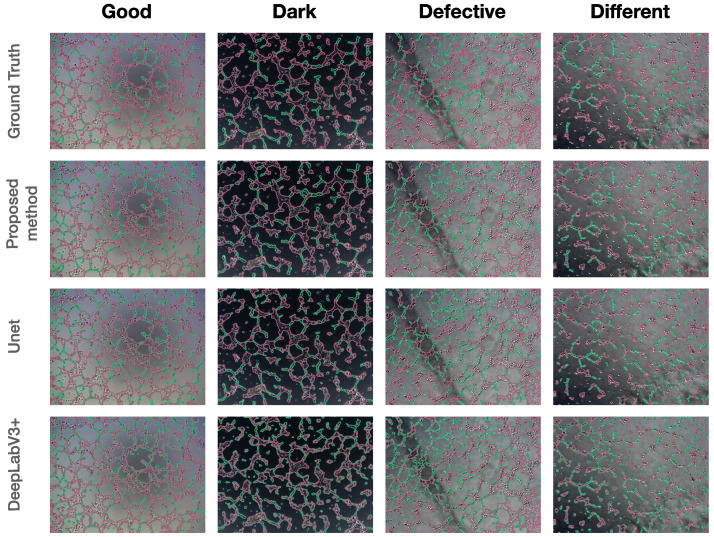
Results of qualitative segmentation by the proposed method, Unet, and DeepLabV3+ (×100 magnification).

Three architectures were considered for the qualitative results: DeepLabV3+, Unet, and our proposed method (Figure 5). We would like to note that DeepLabV3+ had significant drawbacks. It demonstrated a wrong understanding of the layout of objects in photographs and their division into “nodes” and “tubes”. Almost all structures in the photographs were identified as tubes, even single cells and cell debris. Quite often, on cellular structures after marking, you can see that the model labelled a thin area near the nodes as tubes, while this area did not fall under the “tube” category. This defect in the model was called “double marking”. All this meant that DeepLabV3+ did not recognize or categorize objects. In addition, this model did not select objects along the contour and did not repeat their shape, which affected the final numerical data on the length and size of the objects of interest to us.

Compared to DeepLabV3+, the other models were better at understanding and separating objects in photographs into “nodes” and “tubes”. This is evident due to the absence of “double marking”, both as the nodes and tubes of all objects in the photographs. However, each of the models had disadvantages. Unet demonstrated the labelling of single cells both in the structure of the node and in the background of the photographs as tubes; there was also a “double marking” of some structures. Although rare, there was a “double marking”. The proposed method showed the best result. This was evident from the absence of indicating single cells and small groups of cells freely located in the photographs as nodes and the marking of long structures as tubes, as well as single cases of “double marking” and labelling of single cells with constriction as tubes. However, like everything else, this model had a serious drawback—the model did not mark nodes consisting of one cell and located between clusters of tubes.

## 3. Discussion

The process of the formation of new vessels (angiogenesis) is one of the most important processes in human physiology and pathology [7]. Angiogenesis stops in the postnatal period and, under physiological conditions, is limited to the reproductive cycle in women and cyclic processes in the hair follicles. However, without angiogenesis, the repair process is impossible. Angiogenesis underlies many pathological conditions: neoplastic processes, atherosclerosis, diabetes, endometriosis, and diseases associated with chronic inflammation [2,7]. Angiogenesis consists of several stages, each of which is associated with the functional activity of ECs; it is divided into branching and nonbranching. Each type of them has its own characteristics; however, the key stages in the process of vessel formation are the proliferation and migration of ECs [2,5,7].

Angiogenesis has been studied all over the world for a long time [47]. Researchers have been studying both the individual stages of angiogenesis and the entire process using animal and cell models [11,48]. However, the most difficult step in the study of angiogenesis remains the analysis of the obtained data.

Recently, methods and programs have been developed that make it possible to study the processes of ECs’ proliferation and migration at a sufficiently high level and process the data obtained in the course of angiogenesis experiments [48]. The primary methods for assessing proliferative activity are: the assessment of cell number, the detection of DNA synthesis by incorporating labelled nucleotide analogues, the measurement of DNA content, the detection of proliferation markers (KI-67 [49], PCNA [50]), and metabolic assays (MTT assay) [51]. To investigate the migration activity of ECs, the wound healing assay and the transwell cell migration assay (Boyden chamber assay) are used [48,52]. In comparison to proliferation, the estimation of migratory activity is complicated by the fact that the researcher needs to take microphotographs, which must be further processed (calculating the number of migrating cells in the photographs, the area before and after cell migration). In many articles, the authors indicate that ImageJ was used for processing [53,54], and new programs are currently being developed, for example, MarkMigration software (St. Petersburg, Russia) [55].

For a researcher in the field of angiogenesis, experiments to assess the process of the formation of vascular networks are of the greatest interest and complexity. At present, a method using various 3D scaffolds is widely used, an example of which is the matrix Matrigel. However, like any method for assessing the formation of blood vessels, this method is associated with technological difficulties, namely, accounting for the images obtained using a microscope. Often, researchers have to process hundreds of microphotographs to obtain the final result of the experiment, followed by further statistical processing of the data. To process photographs, researchers use ImageJ [54,56] and the AxioVision image analysis system [57] to measure the length and number of tubes, but this is a very time-consuming and labour-intensive process. In addition, a significant problem for researchers of angiogenesis is the processing of photographs obtained during the experiment. Currently, the use of time-lapse microscopy is a routine method. However, taking the multitude of photographs obtained at different stages of the experiment into account remains a difficult task, as, in the process of vessel formation, tubes alter their morphology, size, and branching. Currently, there are a number of imaging systems with integrated software that allow the processing of images with capillary-like structures, such as the Operetta High Content Imaging System by PerkinElmer and the CellInsight CX7 HCA Platform by Thermo Scientific. However, these systems have a number of disadvantages, including the high price of the systems and the need to conduct experiments with fluorescent dyes, which increases the cost of research. Additionally, not all systems allow you to mark several objects at once, in particular tubes and nodes. Thus, it is necessary to create automated systems that allow the processing of angiogenesis photographs, namely, to identify various objects in photographs, to issue numerical data, and to be able to perform statistical analysis.

We developed and validated a fully automated pipeline to analyse microscope-derived ECs images. We used a pretrained EfficientNet-B7 encoder to build a Unet++ deep learning model and applied postprocessing steps to obtain quantities of angiogenesis in vitro. The semantic segmentation model obtained in the series of experiments described in Section 2.1, Section 2.2, Section 2.3 and Section 2.4 showed its accuracy in the average macroscopic index IoU${}_{3}=65.93\pm 0.11$% for three classes and IoU${}_{2}=89.77\pm 0.15$% for two classes. The visualization (Section 2.5) showed that a lot of areas where human and computational predictions diverged were primarily due to the entanglement between the tube and the node, as well as the segmentation of single cells with no important qualities in the background, which was not deliberately marked up by the participants.

To the best of our knowledge, the study is the first to explore deep-learning-based strategies for object segmentation. The main advantage of our method is that the sensitivity of the assay does not depend on image quality, which allows for more consistent results compared to existing methods of image analysis of the angiogenesis process. Indeed, images taken with a microscope do not have to be “perfect” for a particular method. For example, images may have a low contrast, the quality of which is affected by several factors, including the settings of the microscope used to take the image [18].

To train the model, we collected 275 annotated images taken from an AxioObserver Z1 microscope at a 100× magnification (phase contrast). It is the first angiogenesis process dataset publicly available, as far as we know. We divided the dataset into four categories (Good, Dark, Difficult, and Different) based on image quality and content. We suppose this subdivision will help obtain a flexible and more predictive model in future studies. The agreement coefficient showed that the annotated images generated by the participants were not perfect but robust enough to be used as ground truth masks. The annotation protocol was created during the discussion, after which the quality of the resulting masks increased from weak in Phase 1 to close to perfect in Phase 2, and moderate in Phases 3 and 3${}^{*}$. It is up to the researcher to create the model and decide on the use of the data. In addition, we demonstrated that there was no significant difference in the quality of markup between students and experts.

Our annotated dataset is a step that brings us closer to the use of more advanced methods for image analysis of the complex process of angiogenesis. Our approach allows the image analysis to produce quantitative data, which will save experts from inefficient and time-consuming work [58]. Image segmentation followed by skeletonization yielded ten network structure parameters, including branches, closed networks, nodes, network areas, network structures, triple-branched nodes, quad-branched nodes, total branch length, average branch length, and the branch-to-node ratio. Among other things, the uniqueness of our approach is defined by the division of objects formed by ECs into two categories, namely nodes and tubes, which in turn allowed us to obtain parameters such as tube length, tube coverage area, and node area (Supplementary Figure S1).

The definition and division of objects in photographs into nodes and tubes and the determination of their length and area has a number of important functional characteristics. Firstly, by the number, area and length of nodes and tubes formed by cells, and by the number of branches from a particular node, the researcher can determine the type of angiogenesis and the mechanism by which the tubes were formed. Secondly, the researcher can establish the functional activity of ECs and, for example, understand the migration potential of cells depending on specific conditions. Thirdly, the establishment of the area and size of the tube may be useful for further study to determine the lumen in the vessel. The determination of the node area can allow the researcher to understand the correctness of the experiment and the influence of conditions on the functional characteristics of ECs, including their proliferation and migration. For example, the presence of a large number of large-area nodes and short tubes along the well edges may indicate that the researcher made technical errors, such as when layering Matrigel or adding cells to wells. In addition, the ability of the system to identify tubes and nodes separately is very important for processing photographs of various stages of angiogenesis, as it will allow the recording of new nodes, points of formation and branching of vessels, as well as their growth during the experiment. Finally, by having a large number of different parameters and data available and knowing the history of the influence of one or another factor on angiogenesis, it is possible to predict the behaviour of cells in culture and in the process of angiogenesis.

The implementation of this approach enables the analysis of large image sets from time-lapse microscopy, which, in turn, will enhance the mechanistic evaluation and improve functional indices of angiogenesis (including pictures of different stages of angiogenesis) and other biologically important branching processes, e.g., the formation of biological neural networks. Moreover, it paves the way for obtaining the necessary data to determine the kinetics of vascular formation, quantify the rate of network formation and stabilization, and understand the potential mechanisms underlying vascular dysfunction. In the future, these data can be used to create predictive models both for the fundamental study of the mechanisms underlying angiogenesis under normal and pathological conditions and for various test systems for which immediate data acquisition is important.

## 4. Materials and Methods

### 4.1. Dataset Description

A unique dataset consisting of 275 photographs capturing the process of blood vessel growth was used in this study. Preliminary experiments were undertaken to grow vessels from endothelial cells:

ECs of the EA.Hy926 cell line were used (ATCC, Manassas, VA, USA). They reproduced all basic morphological, functional, and phenotypic characteristics of ECs [59,60,61]. The cells were cultured according to the manufacturer’s protocol (ATCC, Manassas, VA, USA). The cells were subcultured every 3–4 days by a 5 min treatment with Versene (BioloT, St. Petersburg, Russia). All cells and experiments with cell culturing were performed in conditions of a humid atmosphere at 37 °C and 5% CO${}_{2}$. Cellular death percentage was evaluated by trypan blue dye (Sigma, Aldrich Chem. Co., St. Louis, MO, USA) inclusion. Cell viability in all experiments was ≥96%. To assess the formation of tubelike structures by EA.Hy926 cells, the wells of a 24-well plate were pretreated with a Matrigel Growth Factors Reduced matrix (BD, Franklin Lakes, NJ, USA). Matrigel, a secretion product of mouse sarcoma cells of the Engelbreth-Holm-Swarm line, represents a mixture of extracellular matrix proteins, such as laminin and type IV collagen and also contains comparatively minor levels of TGFβ, EGF, IGF, bFGF, and PA [62]. In the wells of a 24-well plate, 400 μL of DMEM/F-12 and 25 μL of fetal calf serum (FCS) were added. Then, $1.5\times 10^{5}$ ECs (EA.Hy926 cells) in 500 μL of DMEM/F-12 were added to each well (Sigma-Aldrich Chem. Co., St. Louis, MO, USA). The results of ECs culturing in the presence of 2.5% FCS (BioloT, St. Petersburg, Russia) were taken as a baseline (the length and number of tubes: 99.00 (85.00; 118.47) μm and 115 (104; 122)) and the results of ECs culturing in the presence of 10% FCS were used as a positive control (the length and number of tubes were 113.78 (90.49; 141.24) μm and 197 (181; 209), *p* < 0.001). The plates were incubated for 24 h (37 °C, 5% CO${}_{2}$). The experiments were repeated twice in 3 iterations for each position. In most of the experiments, the obtained data were recorded at the endpoint after 24 h; in some experiments, data were recorded at the endpoint after 10 h (the length and number of tubes were 76.5 (63.66; 96.82) μm and 58 (49; 69), *p* < 0.001) to follow the process of vessel formation. In each well of the plate, photographs of five random visual fields were taken by the AxioObserver Z1 microscope (Carl Zeiss, Oberkochen, Germany) at a 100× magnification (phase contrast). All angiogenesis experiments have been described and published previously [57,63,64,65]. Thus, as a result of the experiments, 275 micrographs were obtained and selected for further analysis using a neural network.

**Figure 6:**
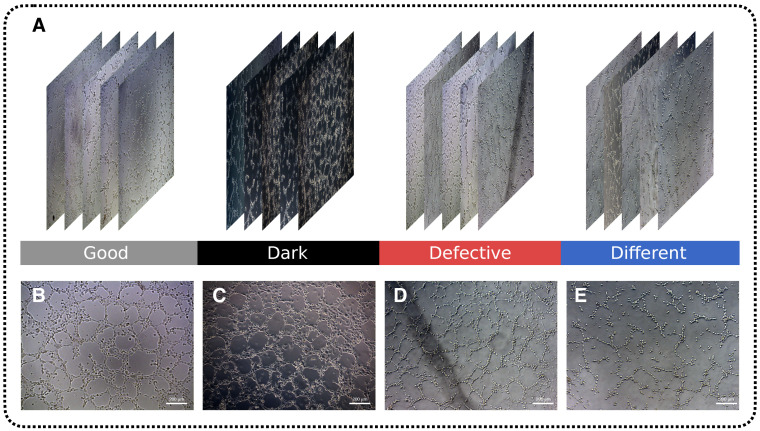
Original dataset consisting of 4 image categories (**A**) Good, Dark, Defective, and Different. (**B**) Image of good quality, easily recognisable structures; ECs were incubated for 24 h. (**C**) Image with altered light, darker than normal; ECs were incubated for 24 h. (**D**) Image with extraneous objects, such as shadows and defocus; ECs were incubated for 24 h. (**E**) Image from the early stages of angiogenesis; ECs were incubated for 10 h. Phase contrast, 100×.

The selected images were divided into 4 categories as shown in Figure 6: “Good”—photos of good quality, convenient for marking and training model, “Dark”—images obtained in experiments with altered illumination due to replaced microscope glass, “Defective”—images with foreign objects against the background, shadows and defocusing, “Different stages”—a few photos in which the process of vessel formation was not completed, for example, when tubes were not fully formed and were not closed into nodes. The last category of images was the most difficult to assess, since it was not obvious when the formed entity could be considered a full-fledged structure in the form of a node or tube, and when it could not. Images were used to mark up incoming data for further training.

### 4.2. Participant Training and Data Collection

**Figure 7:**
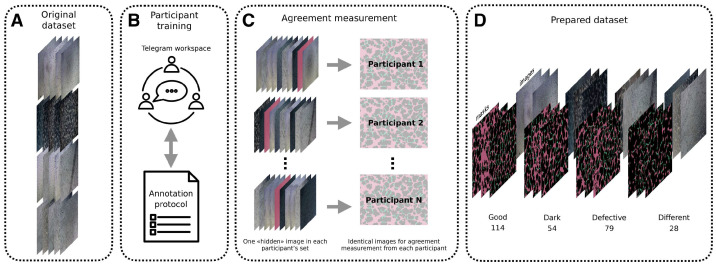
Illustration of workflow. (**A**) The original dataset. (**B**) Labelling was conducted using the online service CVAT with communication through Telegram. During the discussion, the annotation protocol—a set of rules describing the definitions of tubes and nodes for marking—was formed. (**C**) During the data-labelling process, the agreement coefficient was measured periodically to verify that the obtained data were correct. Each member of the labelling group had one identical “hidden” image periodically added to the set of 10 images, and then the labelling of that image from all participants was compared. (**D**) The result was a dataset with a total of 275 images.

The study workflow is illustrated in Figure 7. There were two groups of people involved in the labelling process: students (S) whose field of study was not related to angiogenesis and experts (E) with knowledge of the subject. New participants were added over the course of the project and the number of people in the project varied periodically.

**Figure 8:**
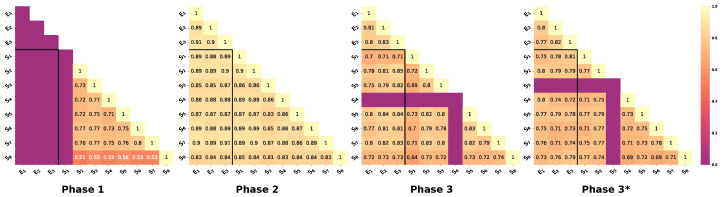
Cohen’s kappa pairwise matrix between students (S) and experts (E) for 4 phases of marking: (**Phase 1**): shared image from the Good group, prior to the creation of the annotation protocol, the markup was independent—participants did not confer, $\bar{\kappa }=0.69\pm 0.10$. (**Phase 2**): shared image from the Good group, immediately after creation of the annotation protocol, participants conferred, $\bar{\kappa }=0.87\pm 0.02$. (**Phase 3**): shared hidden image from the Good group during the labelling process, independent mark-up, $\bar{\kappa }=0.77\pm 0.05$. (Phase 3*): the same conditions as in Phase 3, but with a hidden image from the Different group, which was more difficult to analyse, $\bar{\kappa }=0.75\pm 0.03$. Purple cells without text show that the image was not marked by a particular member of the annotation group. The solid black line divides student–student, expert–expert, and student–expert pairs.

The training of the annotation group was organised. Phase 1 was an initial consultation: an explanation by experts of the general rules about the difference between tubes and nodes for students, as well as instructions for working with the CVAT (Computer Vision Annotation Tool) service for annotation for all participants. CVAT is a computer vision open-source tool for interactive video and image annotation [66]. The interface is convenient for users because it has a web working mode and is compatible with teamwork. The primary functions of the tool are: object detection, image classification, and segmentation. For our purposes, the last one was the most important. For participants’ training, the images were taken from the original dataset (Figure 7A), as well as for the following data collection. Next, each student was given two images: one identical Good image for further agreement coefficient measurement to determine the similarity of the markings (Figure 8. Phase 1, see Section 4.3 for more details), and one individual Defective image for their own practice in labelling. The mark-up process was conducted independently—students did not consult with each other. This was followed by a discussion of their errors with experts: both general ones in the Good image, and more difficult ones in the Defective image. As a result, during the discussion, an annotation protocol was formed: a set of rules according to which nodes and tubes were marked (Supplementary Annotation Protocol (Schemes S1 and S2)). Special attention was paid to the protocol, as many points in the mark-up of structures were not obvious.

In the Phase 2, another pair of images was uploaded for each participant, one of the images was Good, the second was Defective; the comparison was made from the Good image, the second image was added for participants’ practice only. During the labelling process, the participants conferred among themselves and discussed controversial points within their social net workspace (Figure 7B). In addition, participants were actively using the rules from the annotation protocol. Due to the improvement of the agreement coefficient for Phase 2 in comparison with Phase 1 (0.86±0.02 vs. 0.69±0.10, Table 2), it was decided that the training of the participants was successful and it was possible to start preparing the dataset.

After that, the final Phase 3 began: obtaining labelled images for the dataset. In total, each participant was given 3 sets of 10 images. Each set contained a different number of various types of images. An interim measurement of the coefficient of agreement was also periodically carried out to make sure that the annotated data were correct. The participants did not confer and used the annotation protocol. The same images for agreement measurement were mixed into the set of each of the participants in different places in the sequence (Figure 7C). In this phase, participants were unaware that a comparison of images was being made, unlike in the first and second phases. These images were “hidden”.

As a result of the labelling process, the number of image–mask pairs was 275, as shown in Figure 7D: Good—114, Dark—54, Defective—79, Different—28. In the evaluation, all the images were randomly split into two sets: 68% for training and 32% for testing. As shown in Table 1, the training set consisted of 77 Good, 36 Dark, 53 Defective, and 19 Different images annotated and sampled for building AI models.

### 4.3. Measuring Annotation Interparticipant Agreement

As already mentioned above, during the training of the participants of the mark-up group and the dataset creation, the agreement coefficient was measured periodically: first in the training stage to make sure that the participants understood the mark-up rules, during the dataset data collection to monitor the correctness of the resulting data, and also to compare the quality of markings.

Phase 1: after an initial consultation from the experts, the students were given two images, one from the Defective group and one from the Good group, with the image from the Good group being used to measure the agreement coefficient. The purpose of this was to analyse how fine the consent was between participants, and also to discuss errors and subsequently form the annotation protocol, a set of more explicit rules for marking angiogenesis images. Students marked up the images without consulting each other. The mean agreement was: 0.69±0.10.

Phase 2: after the annotation protocol was created, all participants were again given two new images, one from the Defective group and one from the Good group. Participants were able to confer by discussing difficult aspects and also used the annotation protocol. The agreement coefficient was again measured on the Good image. The average agreement was: 0.87±0.02. It is worth noting that this time the students’ agreement was much higher, especially for those participants whose scores were lower than the others in Phase 1. In addition, the mark-up of the experts and the mark-up of the students matched well. Finally, the quality of the markup and the value of the agreement between the participants led to the conclusion that it was possible to start labelling images for dataset.

Phase 3: As previously mentioned, once the process of obtaining the dataset had begun, the agreement coefficient was measured twice: once on the image of the class Good and a second time on the image of the class Different (in Figure 8 Phase 3 and Phase 3*, respectively). At this phase, participants were unaware that a comparison was taking place, and the images were deliberately shuffled into sets of photographs at different locations in the sequence. It should be mentioned that in the process of obtaining the prepared marked images for training the network, the quality was still at a high level, although lower than in Phase 2. This is explained by the fact that the participants were discussing errors with each other, whereas in Phase 3 they were already marking independently. The average agreement in Phase 3 was 0.77±0.05 and in Phase 3* it was 0.75±0.03. Although marking photos from the different phases was much more difficult, it did not greatly affect the quality of agreement between participants: the agreement for both images matched within one standard deviation (more details in Table 4). This demonstrated that the quality of the received images was satisfactory, and that the annotation protocol worked effectively for difficult cases too.

Pairwise interparticipant agreement was measured using Cohen’s kappa [67] (kappa) statistic. The general form of the equation can be written as:

(3) $$\kappa =\frac{p_{0}-p_{e}}{1-p_{e}}$$

where $p_{0}$ denotes the observed probability of agreement, and $p_{e}$ denotes the probability of the expected agreement due to chance. Possible values for κ statistics range from −1 to 1, with 1 indicating perfect agreement, 0 indicating completely random agreement, and −1 indicating “perfect” disagreement [68]. Landis and Koch [69] provided guidelines for interpreting kappa values, with values from 0.0 to 0.2 indicating a slight agreement, 0.21 to 0.40 indicating a fair agreement, 0.41 to 0.60 indicating a moderate agreement, 0.61 to 0.80 indicating a substantial agreement, and 0.81 to 1.0 indicating an almost perfect or perfect agreement. Nevertheless, we recognise that qualitative cutoffs vary depending on the study methods and research question. In our case, the equation for κ can be written as follows:

(4) $$\kappa =\frac{N\sum_{c=0}^{N_{c}-1}\left|I_{c}\cap J_{c}\right|-\sum_{c=0}^{N_{c}-1}\left|I_{c}\right|\cdot \left|J_{c}\right|}{N^{2}-\sum_{c=0}^{N_{c}-1}\left|I_{c}\right|\cdot \left|J_{c}\right|}$$

where *I* and *J* denote two participants with corresponding masks, composed of *c* binary channels, where $N_{c}=3$ is the number of classes being considered, and N=2584×1936—total number of pixels on an image.

Our analysis compared the impact of experience level and feedback on annotation quality. As advised in [68,70], we used the mean kappa to obtain a final measure for 3 or more participants. For each phase, we measured four average kappa values as in Naumov et al.’s [71] work: the first was averaged over expert–expert pairs (${\bar{\kappa }}_{EE}$), the second over student–student pairs (${\bar{\kappa }}_{SS}$), the third over student–expert pairs (${\bar{\kappa }}_{SE}$), and the fourth over all pairs ($\bar{\kappa }$). The first two values provided information about the agreement between the two groups, while the third value showed to what extent the experts agreed with the students. We tended to use $\bar{\kappa }$ as the final measure of expert agreement. The paired Cohen’s kappa for each pair of experts are shown in Figure 8. The average κ values between the different groups are presented in Table 4. We interpreted the agreement in Phase 1 as weak (mean kappa: 0.77), in Phase 2 as close to perfect (mean kappa: 0.87), and in Phases 3 and 4 as moderate (mean kappa values of 0.77 and 0.75).

### 4.4. Evaluation Model Performance

To calculate the model’s performance, we used a standard measure commonly used to solve the object category segmentation problem, called intersection-over-union (IoU). The original equation for the binary problem can be given as:

(5) $${\mathrm{IoU}}_{c}=\frac{|T_{c}\cap P_{c}|}{|T_{c}\cup P_{c}|}$$

where $T_{c}$ and $P_{c}$ are the two masks of the true label and prediction model for the corresponding *c* binary channels. *c* can take the values {background,tubes,nodes}. In this paper, we used the following two metrics:

(6) $$\begin{array}{c} {\mathrm{IoU}}_{3}=\frac{1}{3}\left[{\mathrm{IoU}}_{background}+{\mathrm{IoU}}_{tubes}+{\mathrm{IoU}}_{nodes}\right] \end{array}$$

(7) $${\mathrm{IoU}}_{2}=\frac{1}{2}\left[{\mathrm{IoU}}_{background}+{\mathrm{IoU}}_{cells}\right]$$

where cells=tubes∪nodes. IoU${}_{3}$ allowed us to understand how well the resulting model differentiated all three classes and also how well it understood the difference between tubes and nodes. IoU${}_{2}$ allowed us to understand how well the boundaries between *cells* and the remaining background were defined.

## 5. Conclusions

Extensive and universal work was demonstrated: a dataset with labelled masks was created from the original angiogenesis images, the correctness of which was verified repeatedly by checking the agreement coefficient between participants using Cohen’s kappa statistic. A neural network model of the Unet++ architecture based on a pretrained EfficientNet-B7 encoder was developed and tested on the data. The quality of the model was improved by optimizing the loss function fitting and the fine-tuning process. The segmentation results obtained with this model were impressive, both in the case of the identification of only two classes (background and cells; IoU${}_{2}=89.77\pm 0.15$%) of objects as well as three (background, nodes, and tubes; IoU${}_{3}=65.93\pm 0.11$%). The use of this model significantly improves the efficiency of angiogenesis data by providing a more convenient and faster method of analysis, as opposed to manual processing. The advantages of this system allow its use for the further determination of the kinetics and mechanisms of vascular formation, which is important for the fundamental study of the angiogenesis process, the study of the influence of various factors, and for creating a predictive model of such structures’ growth (for example, Doppler) and test systems that can be introduced into diagnostics and used for the treatment of pathologies, which are based on the process of vascular formation. However, we believe that creating a more perfect prediction system needs further training with a larger set of micrographs of various stages of angiogenesis.

## Figures and Tables

**Table 1:** Annotated images ratio of dataset for AI training and testing.

	Number of Image–Mask Pairs	
Data Type	Training Set (68%)	Testing Set (32%)
Good	77	37
Dark	36	18
Defective	53	26
Different	19	9
All	185	90

**Table 2:** Performance depending on the model architecture. The training and testing were carried out on 68% and 32% of the whole dataset, respectively.

Performance	DeepLabV3+	Unet	Unet++
IoU${}_{3}$	0.556±0.008	0.635±0.006	0.641±0.003
IoU${}_{2}$	0.837±0.003	0.888±0.006	0.8915±0.0017

**Table 3:** Model performance depends on the experiment.

Performance	Focal Loss	Architecture Selection	Fine-Tuning
IoU${}_{3}$	63.5±0.6%	65.1±0.8%	65.93±0.11%
IoU${}_{2}$	88.8±0.6%	89.6±0.5%	89.77±0.15%

**Table 4:** Arithmetic means of Cohen’s kappa between different marking groups.

Agreement Study	Phase 1	Phase 2	Phase 3	Phase 3*
${\bar{\kappa }}_{SS}$	0.69±0.10	0.86±0.02	0.76±0.05	0.74±0.03
${\bar{\kappa }}_{EE}$	−	0.90±0.01	0.83±0.01	0.80±0.02
${\bar{\kappa }}_{SE}$	−	0.88±0.02	0.78±0.05	0.76±0.03
$\bar{\kappa }$	0.69±0.10	0.87±0.02	0.77±0.05	0.75±0.03

## Data Availability

The data presented in this study are available on request from the corresponding author.

## Supplementary Materials

The are available online at https://www.mdpi.com/article/10.3390/ijms24021102/s1.

## Abbreviations

The following abbreviations are used in this manuscript: AP: annotation protocol; CE: cross-entropy; CNN: convolutional neural network; EC: endothelial cell; E: experts; FCS: fetal calf serum; H: hypothesis; IoU: intersection-over-union; MTT: 3-(4,5-dimethylthiazol-2-yl)-2,5-diphenyltetrazolium bromide; PCNA: proliferating cell nuclear antigen; S: students.

## References

[1] Papetti M., Herman I.M. (2002). Mechanisms of normal and tumor-derived angiogenesis. Am. J. Physiol.-Cell Physiol..

[2] Risau W. (1997). Mechanisms of angiogenesis. Nature.

[3] Folkman J., Shing Y. (1992). Angiogenesis. J. Biol. Chem..

[4] Carmeliet P., Jain R.K. (2011). Molecular mechanisms and clinical applications of angiogenesis. Nature.

[5] Udan R.S., Culver J.C., Dickinson M.E. (2013). Understanding vascular development. Wiley Interdiscip. Rev. Dev. Biol..

[6] Secomb T.W., Pries A.R. (2016). Microvascular plasticity: Angiogenesis in health and disease–preface. Microcirculation.

[7] Carmeliet P. (2005). Angiogenesis in life, disease and medicine. Nature.

[8] Folkman J. (2007). Angiogenesis: An organizing principle for drug discovery?. Nat. Rev. Drug Discov..

[9] Herbert S.P., Stainier D.Y. (2011). Molecular control of endothelial cell behaviour during blood vessel morphogenesis. Nat. Rev. Mol. Cell Biol..

[10] Charnock-Jones D., Kaufmann P., Mayhew T. (2004). Aspects of human fetoplacental vasculogenesis and angiogenesis. I. Molecular regulation. Placenta.

[11] Norrby K. (2006). In vivo models of angiogenesis. J. Cell. Mol. Med..

[12] Ponce M.L. (2009). Tube formation: An in vitro matrigel angiogenesis assay. Angiogenesis Protocols.

[13] Khoo C.P., Micklem K., Watt S.M. (2011). A comparison of methods for quantifying angiogenesis in the Matrigel assay in vitro. Tissue Eng. Part C Methods.

[14] Russ J.C. (2006). The Image Processing Handbook.

[15] Wan X., Bovornchutichai P., Cui Z., O’Neill E., Ye H. (2017). Morphological analysis of human umbilical vein endothelial cells co-cultured with ovarian cancer cells in 3D: An oncogenic angiogenesis assay. PLoS ONE.

[16] Arbelaez P., Maire M., Fowlkes C., Malik J. (2010). Contour detection and hierarchical image segmentation. IEEE Trans. Pattern Anal. Mach. Intell..

[17] Arganda-Carreras I., Fernández-González R., Muñoz-Barrutia A., Ortiz-De-Solorzano C. (2010). 3D reconstruction of histological sections: Application to mammary gland tissue. Microsc. Res. Tech..

[18] Varberg K.M., Winfree S., Chu C., Tu W., Blue E.K., Gohn C.R., Dunn K.W., Haneline L.S. (2017). Kinetic analyses of vasculogenesis inform mechanistic studies. Am. J. Physiol.-Cell Physiol..

[19] Chan L., Hosseini M., Rowsell C., Plataniotis K., Damaskinos S. (2019). HistoSegNet: Semantic Segmentation of Histological Tissue Type in Whole Slide Images. Proceedings of the 2019 IEEE/CVF International Conference on Computer Vision (ICCV).

[20] Punn N.S., Agarwal S. (2020). Inception U-Net Architecture for Semantic Segmentation to Identify Nuclei in Microscopy Cell Images. ACM Trans. Multimed. Comput. Commun. Appl..

[21] Saha M., Chakraborty C. (2018). Her2Net: A Deep Framework for Semantic Segmentation and Classification of Cell Membranes and Nuclei in Breast Cancer Evaluation. IEEE Trans. Image Process..

[22] Zhou Z., Rahman Siddiquee M.M., Tajbakhsh N., Liang J. (2018). Unet++: A nested u-net architecture for medical image segmentation. Deep Learning in Medical Image Analysis and Multimodal Learning for Clinical Decision Support.

[23] Attribution 4.0 International [Internet] (2021). Creative Commons Corporation. https://creativecommons.org/licenses/by/4.0/.

[24] Noh H., Hong S., Han B. Learning deconvolution network for semantic segmentation. Proceedings of the IEEE International Conference on Computer Vision.

[25] Badrinarayanan A., Le T.B., Laub M.T. (2015). Bacterial chromosome organization and segregation. Annu. Rev. Cell Dev. Biol..

[26] Ronneberger O., Fischer P., Brox T. (2015). U-net: Convolutional networks for biomedical image segmentation. Proceedings of the International Conference on Medical Image Computing and Computer-Assisted Intervention.

[27] He K., Zhang X., Ren S., Sun J. (2016). Identity mappings in deep residual networks. Proceedings of the European Conference on Computer Vision.

[28] Honari S., Yosinski J., Vincent P., Pal C. Recombinator networks: Learning coarse-to-fine feature aggregation. Proceedings of the IEEE Conference on Computer Vision and Pattern Recognition.

[29] Iglovikov V., Mushinskiy S., Osin V. (2017). Satellite imagery feature detection using deep convolutional neural network: A kaggle competition. arXiv.

[30] Iglovikov V.I., Rakhlin A., Kalinin A.A., Shvets A.A. (2018). Paediatric bone age assessment using deep convolutional neural networks. Deep Learning in Medical Image Analysis and Multimodal Learning for Clinical Decision Support.

[31] Ching T., Himmelstein D.S., Beaulieu-Jones B.K., Kalinin A.A., Do B.T., Way G.P., Ferrero E., Agapow P.M., Zietz M., Hoffman M.M. (2018). Opportunities and obstacles for deep learning in biology and medicine. J. R. Soc. Interface.

[32] Kaggle Teams Carvana Image Masking Challenge–1st Place Winner’s Interview, (nd). https://medium.com/kaggle-blog/carvana-image-masking-challenge-1st-place-winners-interview-78fcc5c887a8.

[33] Iglovikov V., Shvets A. (2018). Ternausnet: U-net with vgg11 encoder pre-trained on imagenet for image segmentation. arXiv.

[34] Xie Q., Luong M.T., Hovy E., Le Q.V. Self-training with noisy student improves imagenet classification. Proceedings of the IEEE/CVF Conference on Computer Vision and Pattern Recognition.

[35] Li Y., Yu Q., Tan M., Mei J., Tang P., Shen W., Yuille A., Xie C. (2020). Shape-texture debiased neural network training. arXiv.

[36] Lee J., Won T., Lee T.K., Lee H., Gu G., Hong K. (2020). Compounding the performance improvements of assembled techniques in a convolutional neural network. arXiv.

[37] Gao S.H., Cheng M.M., Zhao K., Zhang X.Y., Yang M.H., Torr P. (2019). Res2net: A new multi-scale backbone architecture. IEEE Trans. Pattern Anal. Mach. Intell..

[38] Demšar J. (2006). Statistical comparisons of classifiers over multiple data sets. J. Mach. Learn. Res..

[39] Conover W. (1971). Practical Nonparametric Statistics.

[40] Jadon S. A survey of loss functions for semantic segmentation. Proceedings of the 2020 IEEE Conference on Computational Intelligence in Bioinformatics and Computational Biology (CIBCB).

[41] Asgari Taghanaki S., Abhishek K., Cohen J.P., Cohen-Adad J., Hamarneh G. (2021). Deep semantic segmentation of natural and medical images: A review. Artif. Intell. Rev..

[42] Lin T.Y., Goyal P., Girshick R., He K., Dollár P. Focal loss for dense object detection. Proceedings of the IEEE International Conference on Computer Vision.

[43] Chen L.C., Zhu Y., Papandreou G., Schroff F., Adam H. Encoder-decoder with atrous separable convolution for semantic image segmentation. Proceedings of the European Conference on Computer Vision (ECCV).

[44] Yosinski J., Clune J., Bengio Y., Lipson H. (2014). How transferable are features in deep neural networks?. Adv. Neural Inf. Process. Syst..

[45] Bengio Y., LeCun Y. (2007). Scaling learning algorithms towards AI. Large-Scale Kernel Mach..

[46] Amiri M., Brooks R., Rivaz H. (2019). Fine tuning u-net for ultrasound image segmentation: Which layers. Domain Adaptation and Representation Transfer and Medical Image Learning with Less Labels and Imperfect Data.

[47] Leung D.W., Cachianes G., Kuang W.J., Goeddel D.V., Ferrara N. (1989). Vascular endothelial growth factor is a secreted angiogenic mitogen. Science.

[48] Nowak-Sliwinska P., Alitalo K., Allen E., Anisimov A., Aplin A.C., Auerbach R., Augustin H.G., Bates D.O., van Beijnum J.R., Bender R.H.F. (2018). Consensus guidelines for the use and interpretation of angiogenesis assays. Angiogenesis.

[49] Patel S.P., Bourne W.M. (2009). Corneal endothelial cell proliferation: A function of cell density. Investig. Ophthalmol. Vis. Sci..

[50] Zeng Z., Chen H., Cai J., Huang Y., Yue J. (2020). IL-10 regulates the malignancy of hemangioma-derived endothelial cells via regulation of PCNA. Arch. Biochem. Biophys..

[51] Park J.Y., Kwon B.M., Chung S.K., Kim J.H., Joo C.K. (2001). Inhibitory effect of 2’-O-benzoylcinnamaldehyde on vascular endothelial cell proliferation and migration. Ophthalmic Res..

[52] Markova K., Mikhailova V., Milyutina Y., Korenevsky A., Sirotskaya A., Rodygina V., Tyshchuk E., Grebenkina P., Simbirtsev A., Selkov S. (2021). Effects of Microvesicles Derived from NK Cells Stimulated with IL-1*β* on the Phenotype and Functional Activity of Endothelial Cells. Int. J. Mol. Sci..

[53] Venter C., Niesler C. (2019). Rapid quantification of cellular proliferation and migration using ImageJ. Biotechniques.

[54] Collins T.J. (2007). ImageJ for microscopy. Biotechniques.

[55] Markov A.S., Markova K.L., Sokolov D.I., Selkov S.A. (2019). MARKMIGRATION, Russia; 2019. Registration Certificate No. 2019612366 for Computer Program “MarkMigratio”.

[56] Lee H., Kang K.T. (2018). Advanced tube formation assay using human endothelial colony forming cells for in vitro evaluation of angiogenesis. Korean J. Physiol. Pharmacol..

[57] Sokolov D., Lvova T.Y., Okorokova L., Belyakova K.L., Sheveleva A., Stepanova O., Mikhailova V., Sel’kov S. (2017). Effect of cytokines on the formation tube-like structures by endothelial cells in the presence of trophoblast cells. Bull. Exp. Biol. Med..

[58] Carpentier G., Martinelli M., Courty J., Cascone I. Angiogenesis analyzer for ImageJ. Proceedings of the 4th ImageJ User and Developer Conference Proceedings.

[59] Thornhill M.H., Li J., Haskard D.O. (1993). Leucocyte endothelial cell adhesion: A study comparing human umbilical vein endothelial cells and the endothelial cell line EA-hy-926. Scand. J. Immunol..

[60] Edgell C.J., McDonald C.C., Graham J.B. (1983). Permanent cell line expressing human factor VIII-related antigen established by hybridization. Proc. Natl. Acad. Sci. USA.

[61] Riesbeck K., Billström A., Tordsson J., Brodin T., Kristensson K., Dohlsten M. (1998). Endothelial cells expressing an inflammatory phenotype are lysed by superantigen-targeted cytotoxic T cells. Clin. Diagn. Lab. Immunol..

[62] Benelli R., Albini A. (1999). In vitro models of angiogenesis: The use of Matrigel. Int. J. Biol. Markers.

[63] Belyakova K.L., Stepanova O.I., Sheveleva A.R., Mikhailova V.A., Sokolov D.I., Sel’kov S.A. (2019). Interaction of NK cells, trophoblast, and endothelial cells during angiogenesis. Bull. Exp. Biol. Med..

[64] Markova K.L., Stepanova O.I., Sheveleva A.R., Kostin N.A., Mikhailova V.A., Selkov S.A., Sokolov D.I. (2019). Natural killer cell effects upon angiogenesis under conditions of contact-dependent and distant co-culturing with endothelial and trophoblast cells. Med. Immunol..

[65] Lvova T.Y., Belyakova K.L., Sel’kov S.A., Sokolov D.I. (2017). Effect of THP-1 cells on the formation of vascular tubes by endothelial EA.Hy926 cells in the presence of placenta secretory products. Bull. Exp. Biol. Med..

[66] Sekachev B., Manovich N., Zhiltsov M., Zhavoronkov A., Kalinin D., Hoff B., Tosmanov, Kruchinin D., Zankevich A., DmitriySidnev (2020). Opencv/cvat: v1.1.0. OpenAIRE.

[67] Cohen J. (1960). A coefficient of agreement for nominal scales. Educ. Psychol. Meas..

[68] Hallgren K.A. (2012). Computing inter-rater reliability for observational data: An overview and tutorial. Tutor. Quant. Methods Psychol..

[69] Landis J.R., Koch G.G. (1977). The measurement of observer agreement for categorical data. Biometrics.

[70] Light R.J. (1971). Measures of response agreement for qualitative data: Some generalizations and alternatives. Psychol. Bull..

[71] Naumov A., Ushakov E., Ivanov A., Midiber K., Khovanskaya T., Konyukova A., Vishnyakova P., Nora S., Mikhaleva L., Fatkhudinov T. (2022). EndoNuke: Nuclei Detection Dataset for Estrogen and Progesterone Stained IHC Endometrium Scans. Data.

