# Supplementary material for: Deep Semantic Segmentation of Angiogenesis Images
Source: Int J Mol Sci. 2023 Jan 6;24(2):1102. doi: 10.3390/ijms24021102 (PMC9866671; doi:10.3390/ijms24021102)
Supplement: Supplementary file 1 [file ijms-24-01102-s001.zip › ijms-2137506-supplementary.pdf]

Annotation Protocol

Labelling vessels

3 classes of objects: nodes (red), tubes (green), background+foreign objects — without labelling.

1. **Node** – tight cluster of cells (Scheme S1)
    1.1. Two types of nodes:
        1.1.1. Node with outgoing thin structures
        1.1.2. Node-island
        - If node contains 5 or more cells – label as node
        - If node contains less than 5 cells – label as background

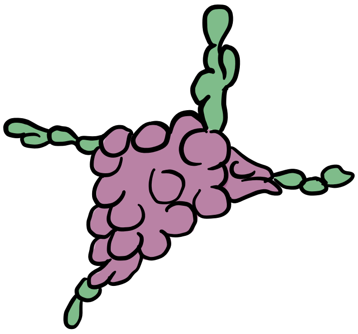

Scheme S1. Node example

2. Connection area
    2.1. Thickness of connection 1-2 cells
    - thin sequence of two or more cells in a row – tube
    - thin row consisting of 1 cell → still node

    2.2. Thickness of connection 2 and more cells → label as node until thickness of structure decreases to 1-2 cells 2 times in a row.If thinning has not occurred - still a node

3. **Tube** – a thin sequence of two or more cells along the length, located side by side. One or two ends attached to the node.
    3.1. The tube may thicken (1-2 cells). If there are 4 or more cells in the thickening (including the cell of the tube) → node (Scheme S2)
    3.2. Tube can bend
    3.3. Another tube cannot come out of one: if the tubes intersect, then the intersection is a node of such thickness that the tubes do not touch each other
    3.4. Single cells next to the tubes are not labelled

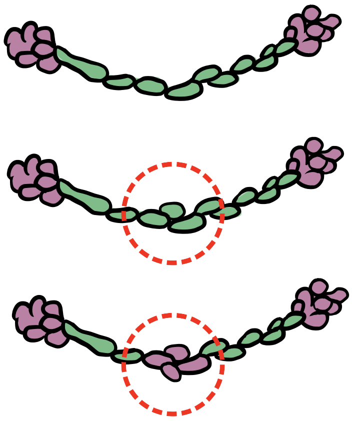

Scheme S2. Tube and node examples

4. Other structures — the structures, which remained after the marking of tubes and nodes, they are not labelled: single cells, tight clusters of 4 or less cells, constrictions — elongated structures (5.2), extraneous objects.

5. Additions
    5.1. Long linear structure labelling: if structure consists of 5 or more cells with visible growth process → label from the larger end of the node and attach a tube to it
    5.2. Constriction labelling: label the constriction as a tube only if it is "tight": it is comparable in thickness to the thickness of the cell and does not disappear. In all other cases, even if there are cells inside it, the constriction is not marked

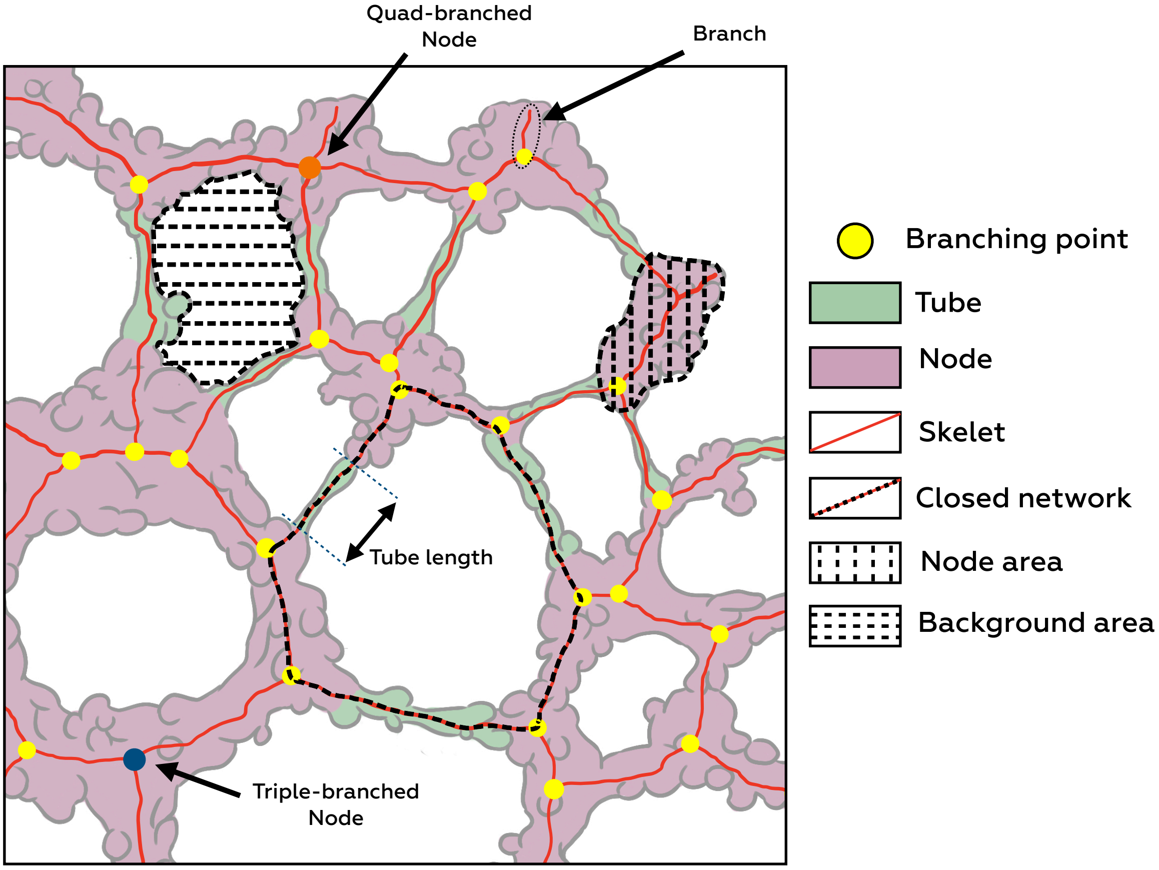

Figure S1. Schematic presentation of the blood vessel network parameters:

1. Branches;
2. Closed networks;
3. Nodes;
4. Network areas;
5. Network structures;
6. Triple-branched nodes;
7. Quad-branched nodes;
8. Total branch length;
9. Average branch length;
10. The branch to node ratio
11. Tube length;
12. Tube coverage area;
13. Node area.

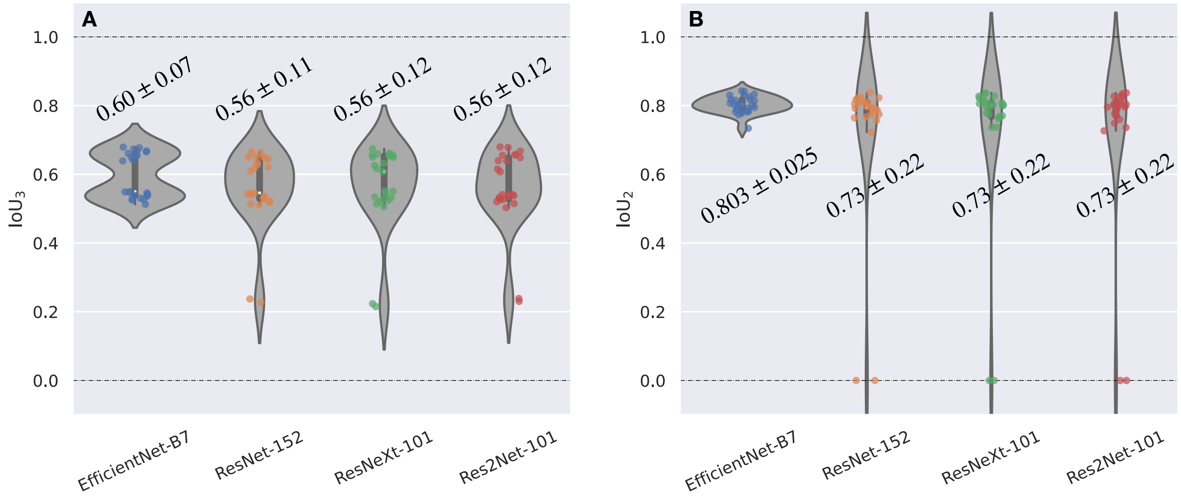

Figure S2. Prediction scores for the validation data; each dot represents performance for *k*-th model out of 25-fold cross-validation; 4 encoders were considered: EfficientNet-B7, ResNeXt-101, ResNet-152, and Res2Net-101. (**A**) Performance by IoU3 metric. The Wilcoxon signed-rank test: median performance of U-net with EfficientNet-B7 greater than for the model with ResNeXt-101, ResNet-152, and Res2Net-101 with a confidence level of 0.95. (**B**) Performance by IoU2 metric. The Wilcoxon signed-rank test: median performance of U-net with EfficientNet-B7 greater than for the model with Res2Net-101, ResNet-152 with a confidence level of 0.95.

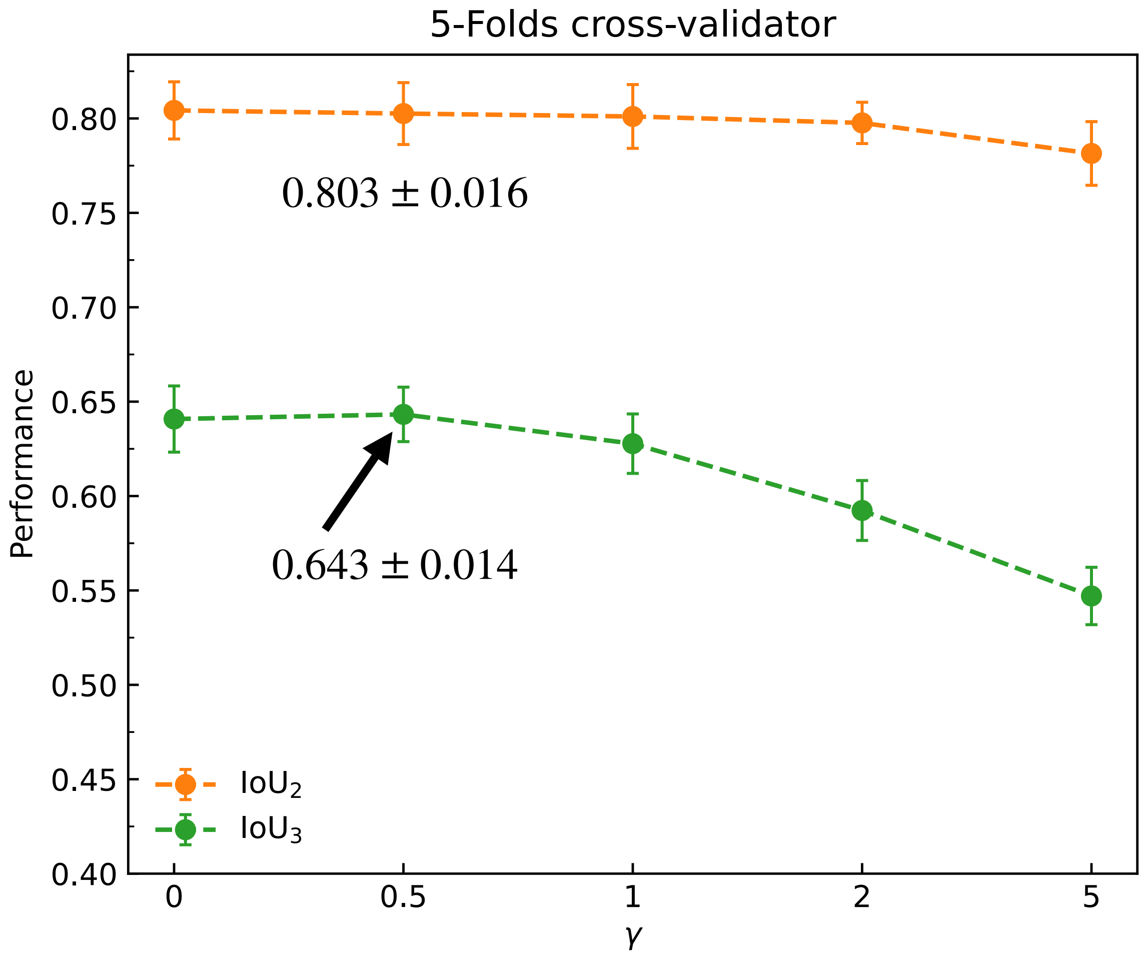

Figure S3. Average performance for different gamma values. We found $\gamma = 0.5$ to work best (by IoU_3) in our experiments. Error bars depict the standard deviation of the mean among the five folds.

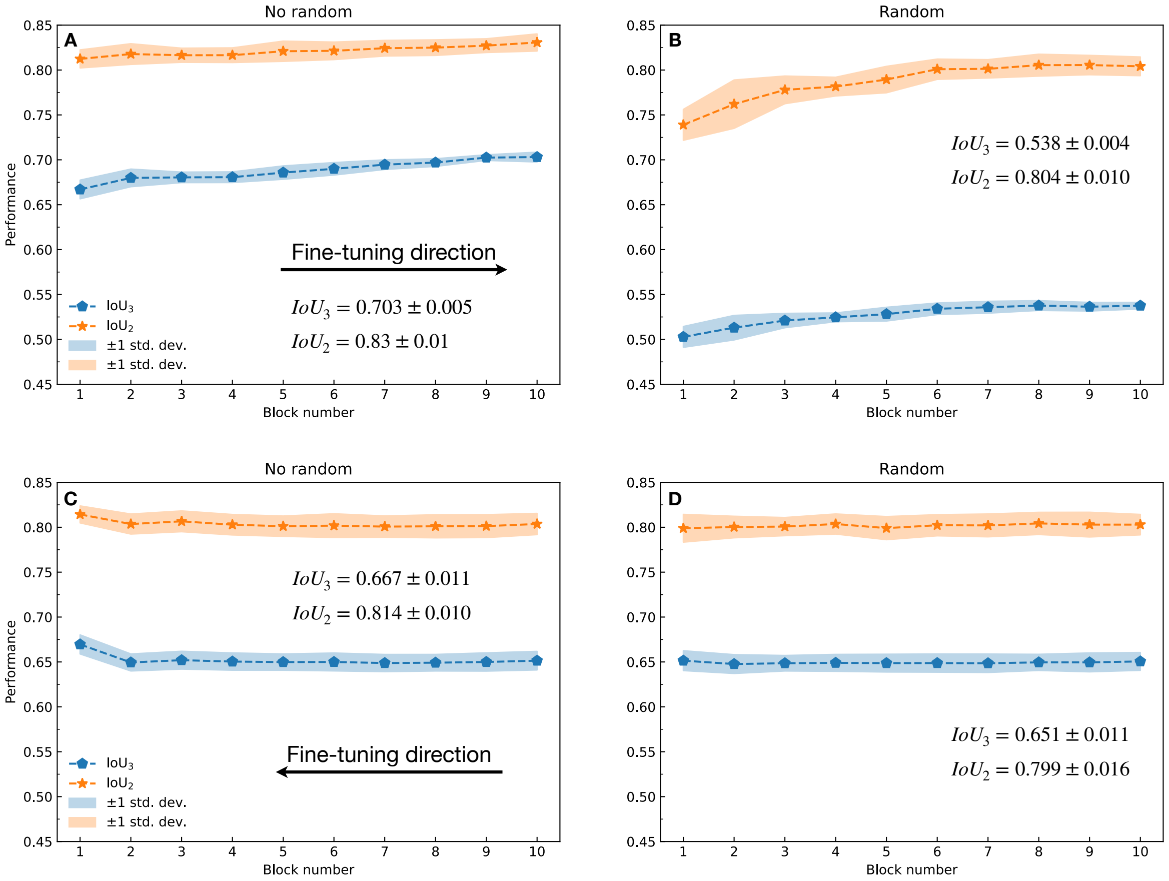

Figure S4. Average IoU_3 and IoU_2 scores depend on the number of trainable blocks in the EfficientNet-B7 encoder. The arrow shows the direction of the fine-tuning blocks: from the first to the last. (**A**) With fine-tuning (forward direction), the block weights are not reset. Archived performance: $IoU_3 = 0.703 \pm 0.005$, $IoU_2 = 0.83 \pm 0.01$. (**B**) With fine-tuning (forward direction), the block weights are reset. Achieved performance: $IoU_3 = 0.538 \pm 0.004$ and $IoU_2 = 0.804 \pm 0.010$. (**C**) With fine-tuning (reverse direction), the block weights are not reset. Archived performance: $IoU_3 = 0.667 \pm 0.011$, $IoU_2 = 0.814 \pm 0.010$. (**D**) With fine-tuning (reverse direction), the block weights are reset. Achieved performance: $IoU_3 = 0.651 \pm 0.011$ and $IoU_2 = 0.799 \pm 0.016$.
